# Epidemic of Chorea Minor

**Published:** 1871-03

**Authors:** 


Epidemic of Chorea Minor.—Dr. Roger, one of the most strenuous advocates of the relationship between rheumatism and chorea (see *Gaz. des Hop.*, 1870, Nos. 65, 66), relates a case of chorea combined with slight articular rheumatism and defect of mitral valve, in which the chronic movements were confined in great measure to the muscles of the right side, while it was on the left side that the rheumatic symptoms were chiefly observed. Dr. Steiner, (*Jhrb. f. Kinderheilk.* N. F., III, 1870) witnessed during the first two months of the year 1870, he tells us, a slight epidemic, nineteen cases, of chorea, the production of which could in no case be referred to imitation, inasmuch as no intercourse between the patients had taken place. As the common cause of the disease, in all the cases, Dr. S. adduces the abnormal condition of the weather at the time the epidemic occurred; its general unusual severity, and the unusually sudden and frequently recurring changes in its temperature; in consequence of which any disorder under which children happened to labor, passed over into inflammation, the result, as Dr. S. supposes, of an irritation set up in the spine. In only five out of the nineteen cases was it possible to detect with certainty rheumatic symptoms—partly articular and partly endocardiac. The remaining fourteen cases, therefore, must be received as those of a rheumatic affection of the spinal meninges, unassociated with any rheumatic inflammation of the muscles of the joints, or of the heart.

In the treatment of the nineteen cases referred to, Dr. S. found the bromide of potassium to be without effect. Fowler's solution in combination with opium proved, as heretofore, particularly beneficial in its effects, whether in cases attended with great restlessness or with a state of entire quietude.—*Centralblatt f. d. Med. Wissenschftn.*, July 9, 1870. D. F. C.

